# Synthesis, characterization and properties of novel polyamides derived from 4 4ʹ bis(4-carboxy methylene) biphenyl and various diamines

**DOI:** 10.1080/15685551.2020.1826705

**Published:** 2020-09-30

**Authors:** Shivaji D. Ghodke, Aslam B. Tamboli, Arati V. Diwate, Vijay P. Ubale, Noormahmad N. Maldar

**Affiliations:** a Arts Science and Commerce College, Osmanabad, MH, India; b Santosh Bhimrao Patil Arts, Commerce and Science College, Mandrup, India; c Sangameshwar College Solapur, India; d D.B.F. Dayanand College of Arts and Science, Solapur, India; e Punyashlok Ahilyadevi Holkar Solapur University, Solapur, India

**Keywords:** 4 4’ bis(4- Carboxy methylene) Biphenyl, polyamides by Yamazaki phosphoryalation method, thermal stability, glass transition temperature

## Abstract

Novel aromatic aliphatic diacid monomer, 4 4ʹ bis(4-carboxy methylene) biphenyl was synthesized via friedel craft acylation with acetic anhydride followed by willgerodot reaction and used in the preparation of polyamides by direct polycondensation using the Yamazaki phosphorylation reaction with commercial aromatic diamines. These polyamides were obtained in good yield with moderate-to-high-molecular-weight build up as evidenced by inherent viscosities in the range of 0.52–0.96 dlg−1. The XRD studies showed that all the polyamides were amorphous in nature and these polymers dissolved in polar aprotic solvents such as N-methyl-2-pyrrolidone(NMP),N,N-dimethylsulfoxide(DMSO), N,N-dimethyl formamide (DMF) and N,N-dimethyl acetamide. The resulting polyamides were characterized by inherent viscocity measurements, FT-IR, 1H NMR and 13C NMR spectroscopy, solubility, thermogravimetry, X-ray diffraction studies. These polyamides had glass transition temperatures between 210°C and 261°C, and their temperatures at a 10% weight loss were 620 to 710°C and 497 to 597°C in nitrogen and air atmospheres, respectively, which indicates thermally stable polymers.

## 1. Introduction

Polyamides are well known for their excellent thermal stability, outstanding mechanical properties and chemical resistance. However, these wholly aromatic polyamides have some drawback and limitations such as poor solubility in some common organic solvents and their extremely high melting temperatures. Most of them do not melt before decomposition because of the rigidity nature and strong intermolecular force of attraction. These properties give rise to difficulties in processing and limitations in their applications. In order to overcome these problems, flexible units such as -NHCO-, -O-, -CH2- and -SO2-, bulky pendent substituents, and non-coplanar moieties were introduced to the polymer backbone to reduce the chain interaction and rigidity nature of the polymer [1–15].

In the past worked polyamides derived from biphenyl unit containing monomer by adding substituent at 2 2ʹ position on biphenyl such as methyl, cyano, phenyl, halogen, sulfonic acid and trifluoromethyl phenoxy groups [16–19]. The bulky phenyl and naphthyl units incorporated at the 2,2ʹ substituted position of biphenylene as pendant groups could reduce the interchain interaction of the polymers, packing efficiency, crystallinity and increases free volume to enhance the solubility of polymers with retention of high thermal stability [20–22].

We have demonstrated an approach to make high-temperature soluble aromatic polymers with sustained thermal stability. We report the synthesis of new novel 4 4ʹ bis(4-carboxy methylene) biphenyl from biphenyl and acetic anhydride starting material via friedel craft acylation followed by willgerdot reaction, it contained methylene spacer group. The polyamides were prepared via direct Yamazaki phosphorylation reaction with commercial aromatic diamines and their properties were compared with those polymers prepared from 2 2ʹ substituent biphenyl. Prepared polyamides containing methylene spacer group in there backbone to improve the solubility, thermal properties and glass transition temperature. Methylene spacer group reduces the chain–chain interaction and intermolecular force of attraction.

The structure of prepared aromatic diacid was confirmed by infrared (IR) and nuclear magnetic resonance spectroscopy (NMR) and the prepared polyamides were characterized by IR spectroscopy, inherent viscosity, solubility, thermal analysis and XRD analysis. The influence of the introduction of methylene spacer groups on the solubility and processability of aromatic polymers has been studied.

We have reported polymers derived from 4 4ʹ substituent biphenyl unit with improved solubility and the retention of high thermal stability than polymers derived from 2 2ʹ substituent biphenyl unit. The attachment of methylene spacer group at 4 4ʹ position on biphenyl decreases polymer interchain interaction to reduce packing efficiency and crystallinity leads to enhanced solubility.

## 2. Experimental

### 2.1 Materials

Acetic anhydride, anhydrous aluminum chloride, biphenyl, carbon disulphide, sulphur, morpholine. N-Methyl-2-pyrrolidone (NMP) were purified by distillation under reduced pressure over calcium hydride and stored over 4Å molecular sieves. Pyridine was refluxed over potassium hydroxide pellets, distilled and stored over 4Å molecular sieves, LiCl was dried under vacuum at 150°C for 6 hrs. Triphenyl phosphite (sigma-Aldrich). 4,4ʹ- Oxydianiline (ODA), 4,4ʹ- sulfonyl dianiline (SDA) 4,4ʹ- methylene dianiline (MDA) were recrystallized prior to use.

#### 2.1.1. Measurements

The FT-IR spectra of organic compounds and polymers were recorded using Nicolet spectrometer. 1H NMR (400 MHz) and 13C NMR (100 MHz) spectra were obtained with a Bruker Advance spectrometer at 25°C using CDCl3 and DMSO- d6 as solvent. Polyamides inherent viscosities were obtained with a polyamide concentration of 0.5 g/dL in DMF solvent at 30°C using an Ubbelhode suspended level viscometer. Differential scanning calorimetry (DSC) was measured on a Mettler Toledo DSC STARe instrument at heating rate of 200°C/min under nitrogen. The glass transition temperatures (Tg) were determined from DSC curves. Thermogravimetric analysis (TGA) was performed on a Mettler Toledo STARe instrument at a heating rate of 10°C/min under nitrogen. Wide angle X-ray diffraction (WAXD) was measured with a Rigaku X-ray diffractometer using polyamide powder.

### 2.2 Diacid synthesis

The new aromatic diacid was synthesized in two steps; as given below.

- Synthesis of 4, 4ʹ diacetyl Biphenyl: In a one litre, three-neck round bottom flask equipped with a pressure-equalizing dropping funnel, a mechanical stirrer and a reflux condenser were placed with 27.72 g (0.18 mol) biphenyl, 280 mL dry carbon disulphide and 120 gm (0.9 mol) of finely powdered anhydrous aluminium chloride. Stir the mixture and heat on oil bath until gentle refluxing commences; add 36.81 mL (0.39 mol) of redistilled acetic anhydride slowly through the dropping (30–50 minutes). The reaction mixture was stirred for 12 h at room temperature and poured in 100 ml beaker containing mixture of ice water. It was filtered and washed with cold ethanol:water mixture (70: 30). Yield 35.6 gm 84%, M. P. 192–194°C.
- Synthesis of 4 4ʹ bis(4-carboxy methylene) biphenyl: In a 100-mL round bottom flask equipped with magnetic stirrer and reflux condenser were placed 21.33 g (0.12 mol) 4, 4ʹ diacetyl biphenyl, 11.66 g (0.36 mol sulphur and 32.33 g (32.33 mL, 0.36 mol) morpholine. The resulting reaction mixture was stirred initially under gentle reflux until the evolution of hydrogen sulphide subsided and then more vigorously for a total period of 14 h at 130°C. The reaction mixture was allowed to cool and poured into 400 mL warm ethanol and left for recrystallisation. 4, 4ʹ biphenyl thioacetomorpholide separated as pale buff crystals; it was filtered and washed with cold ethanol. Yield 35.4 g 89.77%, M. P. 230°C. The product was taken in a 1 L round bottom flask equipped with reflux condenser, magnetic stirrer to which 500 mL of 10% alcoholic NaOH were added. The reaction mixture was refluxed for 5 h, allowed to cool to room temperature and poured into 2 L water. Most of ethanol distilled out under reduced pressure. To the residual product 500 mL, hot water was added and filtered. The filtrate was acidified by 1:1 hydrochloric acid. The precipitated product was filtered, washed thoroughly with hot water and dried. The yield of 4 4ʹ bis(4-carboxy methylene) biphenyl was 15.2 g; 69.98 %, M. P. 150°C. It was recrystallized from aq. ethanol.

FT-IR(KBr, cm−1): 2927 cm−1(-OH), 2854 cm−1 aliphatic – CH,1694.14 cm−1 (Carbonyl)

1H NMR (DMSO, ppm): 3.5 ppm (d, 4 H), 3.5 (d,2 H), 7.2 ppm (d, 4 H), 7.4 ppm (d, 4 H).

13C NMR (DMSO,ppm): 40.93 ppm (CH2), 126, 130, 134, 138 ppm (CH),173 ppm (-COOH)

### 2.3. Polymerization

A mixture of 0.270 g (1 mmol) of 4 4ʹ bis(4-carboxy methylene) biphenyl, 0.200 g (1 mmol) of diamine DA-1, 0.200 g of lithium chloride, 0.63 ml of triphenyl phosphite, 0.5 mL of pyridine, and 2.5 mL of NMP was heated with stirring at 110°C for 3 h under nitrogen atmosphere. After cooling, the reaction mixture was poured into a large amount of methanol with constant stirring, producing a fibrous precipitate that was washed thoroughly with methanol and hot water, collected on a filter, and dried at 110°C under vacuum for 24 h. The inherent viscosities in the range of 0.52 to 0.96 dlg−1. The IR spectrum (film) exhibited absorptions at 1654.45 cm−1 (C = O),2918.73 cm−1 aliphatic – CH and 3279.45 cm−1(-NH stretch of amide). All the other PAIs were prepared using a similar procedure.

## 3. Result and discussion

The aim of this study was to focus on improving the solubility of polyamides and thermal stability. We have been synthesized a series of aliphatic aromatic polyamides containing methylene spacer group in the main chain of polymer backbone by Yamazaki’s phosphorylation method to improve the solubility and inherent viscosity of these polyamides. These aliphatic aromatic polyamides showed inherent viscosities in the range of 0.52–0.96 dl g−1 as shown in Table 1 and good solubility in polar aprotic solvent such as N-methyl-2- Pyrrolidone(NMP), dimethylsulfoxide(DMSO), N,N- dimethyl formamide (DMF) and N,N-dimethyl acetamide. Enhanced solubility of these aromatic polyamides may be due to the methylene spacer group into the polymer backbone. These polyamides had glass transition temperatures between 210°C and 261°C, and their temperatures at a 10% weight loss were 620 to 710°C and 497 to 597°C in nitrogen and air atmospheres, respectively. The synthesized polyamides and 4 4ʹ bis(4-carboxy methylene) biphenyl was characterized by TLC FT-IR, NMR (1H, 13C) and elemental analysis.

**Table 1.** Solubility of polyamides

Polymer → solvent ↓	PA-1	PA-2	PA-3	PA-4	PA-5
DMAc	+ +	+ +	+ +	+ +	+ +
NMP	+ +	+ +	+ +	+ +	+ +
DMSO	+ +	+ +	+ +	+ +	+ +
DMF	+ +	+ +	+ +	+ +	+ +
m-Cresol	+ +	+ +	+ +	+ –	+ –
CHCl3	+ +	+ –	+ +	+ –	+ –
DCM	+ –	– –	+ –	– –	– –
H2SO4	+ +	+ +	+ +	+ +	+ +

+ +: soluble, + –: partially soluble, – –: insoluble.

### 3.1 Diacid synthesis

**Scheme 1.**
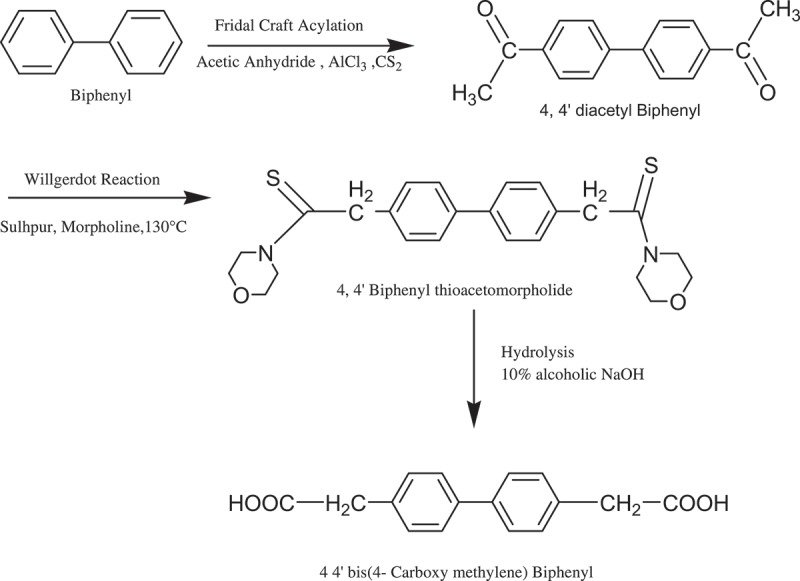
Synthesis of 4 4ʹ bis(4-carboxy methylene) biphenyl monomer

The 4 4ʹ bis(4-carboxy methylene) biphenyl monomer was synthesized in two steps (Scheme 1). In the first step, 4 4ʹ diacetyl biphenyl was prepared from biphenyl and acetic anhydride in the presence of lewis acid catalyst anhydrous aluminum chloride, which undergoes fridal craft acylation reaction on biphenyl. In the second step, 4 4ʹ bis(4-carboxy methylene) biphenyl was prepared from willgerdot reaction of 4 4ʹ diacetyl biphenyl (scheme 1).

Scheme 1. Synthesis of 4 4ʹ bis(4-carboxy methylene) biphenyl monomer.

### 3.2 Characterization of diacid

**Figure 1.**
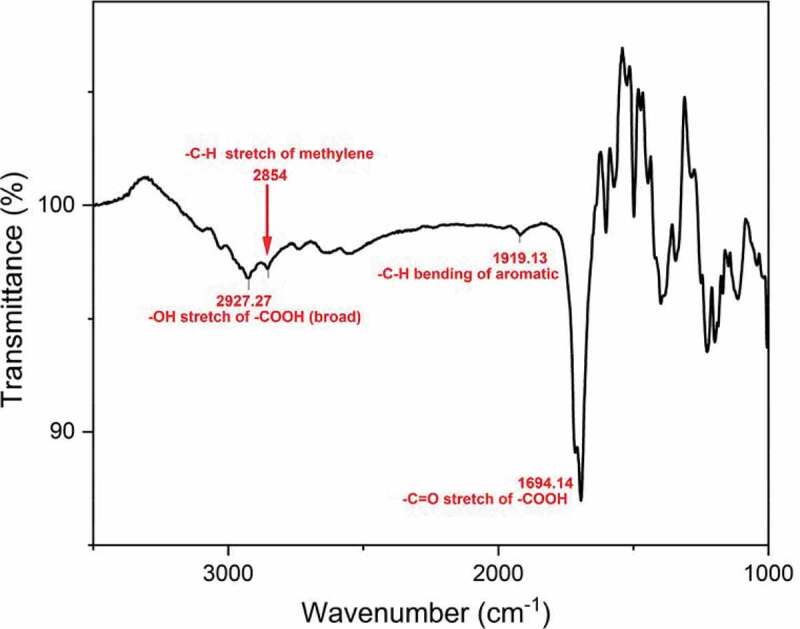
FT-IR spectrum of 4 4ʹ bis(4-carboxy methylene) biphenyl

**Figure 2.**
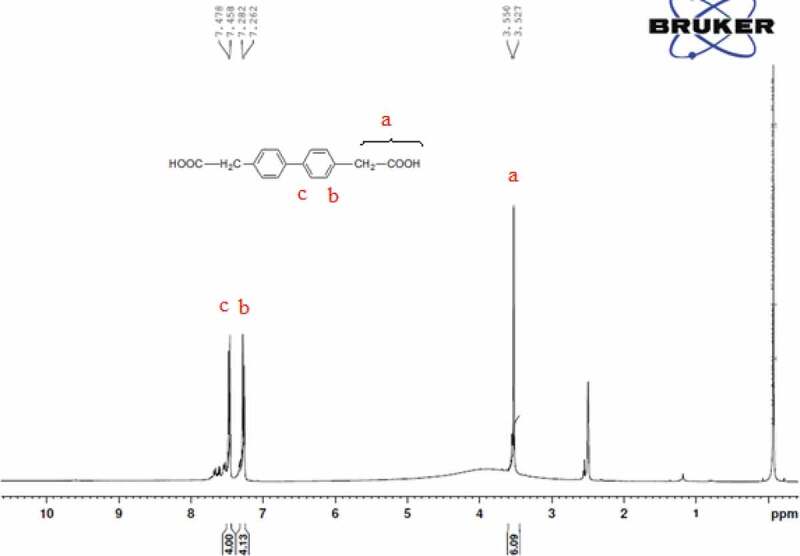
1H NMR spectrum of 4 4ʹ bis(4-carboxy methylene) biphenyl

**Figure 3.**
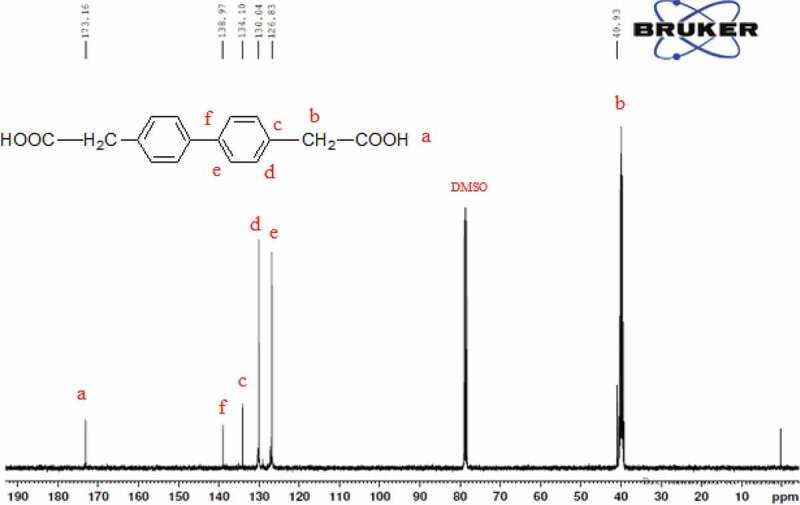
13C NMR spectrum of 4 4ʹ bis(4-carboxy methylene) biphenyl

**Figure 4.**
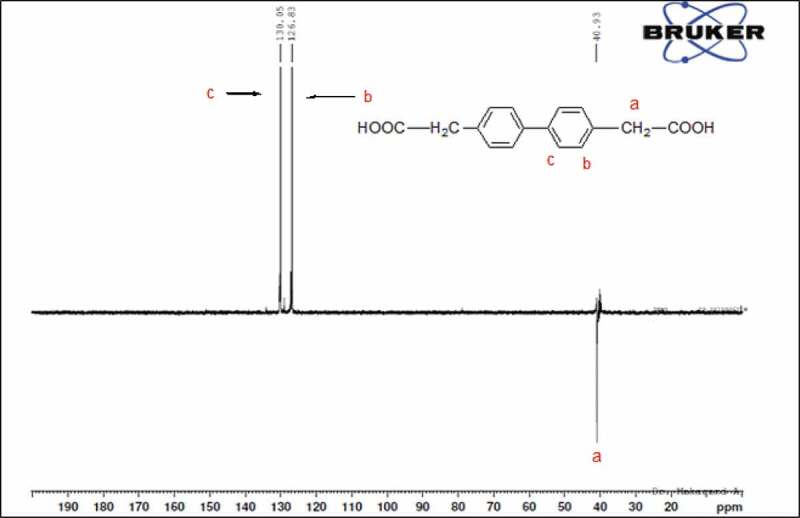
DEPT 13C NMR spectrum of 4 4ʹ bis(4-carboxy methylene) biphenyl

The chemical structure of 4 4ʹ bis(4-carboxy methylene) biphenyl monomer, 4 4ʹ bis(4-carboxy methylene) biphenyl was confirmed by FT-IR and NMR (1H 13C). FT-IR spectrum of 4 4ʹ bis(4-carboxy methylene) biphenyl showed two characteristic stretching absorption band at 2927.27 cm−1 due to the hydroxyl group (–OH) of carboxylic acid, 2854 cm−1 due to the aliphatic – CH stretching and absorption band at 1694 cm−1 due to the carbonyl stretching as shown in Figure 1. 1H spectra of 4 4ʹ bis(4-carboxy methylene) biphenyl the characteristic signal of methylene C-H proton at 3.5 ppm. However, the proton for the carboxylic acid group of 4 4ʹ bis(4-carboxy methylene) biphenyl in the 1H-NMR spectrum was not observed. This was probably due to the fast exchange of this proton with the trace amount of moisture associated with the solvent, whereas the characteristic signal at 7.3 and 7.4 ppm is due to aromatic protons as shown in Figure 2. The 13C spectrum of 4 4ʹ bis(4-carboxy methylene) biphenyl showed six different signals corresponding to six nonequivalent carbons. The signal at 40.93 ppm is assigned to – CH2 group and it is confirmed DEPT 13C NMR spectrum, whereas peaks at 126.83, 130.04, 134.10 and 138.97 ppm due to the aromatic protons and the signal at 173.16 ppm correspond to the carboxylic carbon. This was confirmed by 13C NMR spectrum and DEPT as shown in Figures 3 and Figures 4.

**Scheme 2.**
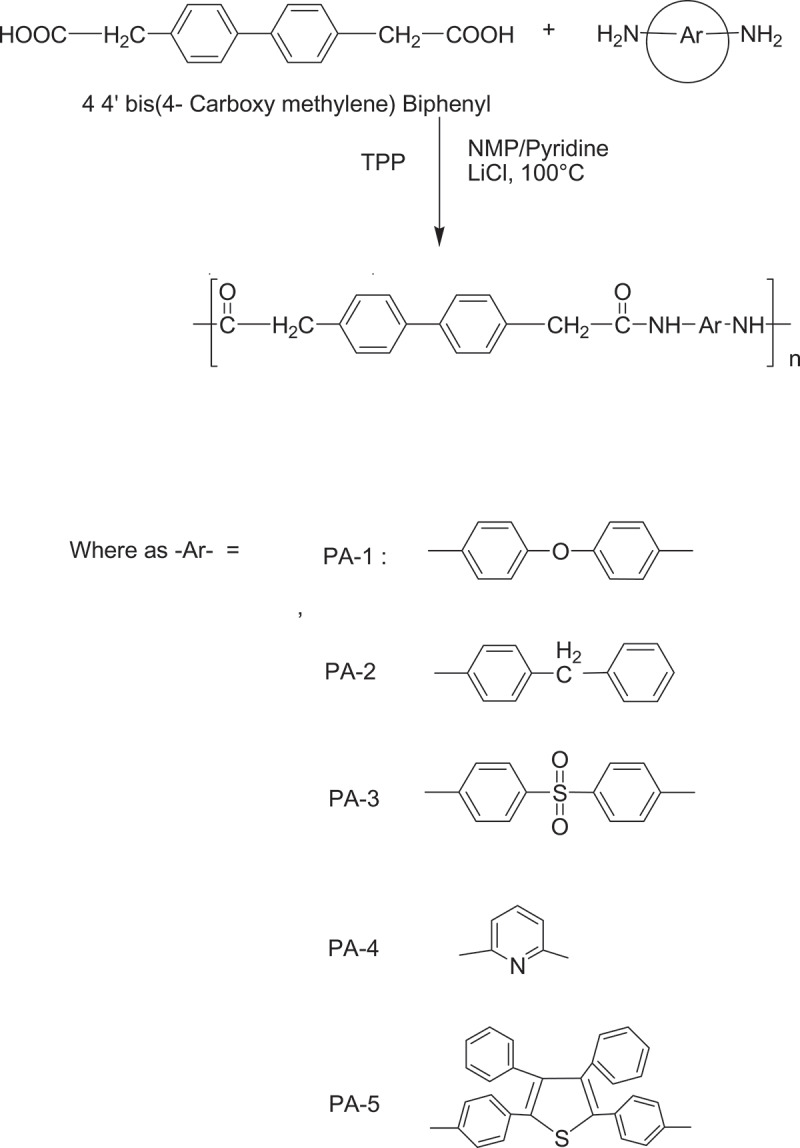
Synthesis of polyamides from 4 4ʹ bis(4-carboxy methylene) biphenyl and various aromatic diamines by Yamazakiʹs phosphorylation method

### 3.3 Polymer synthesis

**Figure 5.**
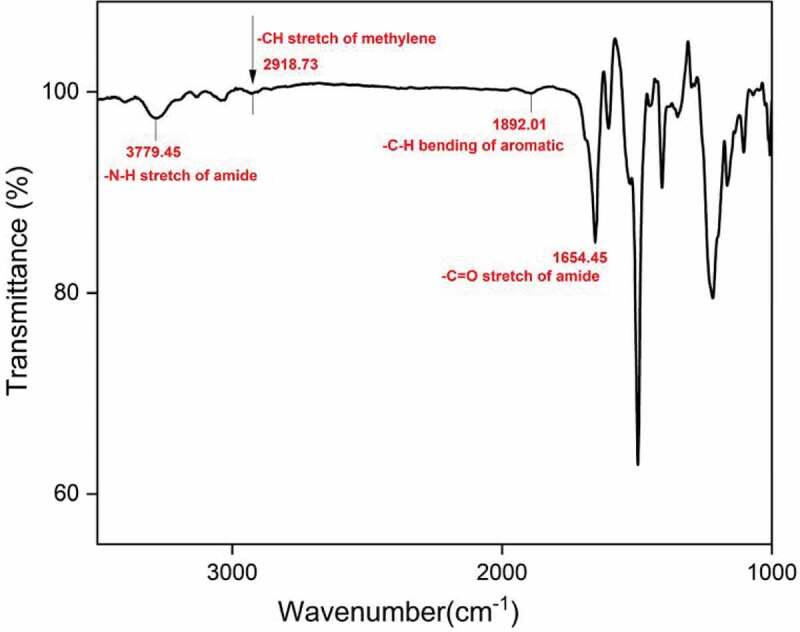
FT-IR spectrum of PA – I

The aliphatic aromatic polyamides were prepared by direct polycondensation of 4 4ʹ bis (4-carboxy methylene) biphenyl with various aromatic diamines in NMP by the Yamazaki et al. method using TPP and Pyridine as condensing agents (scheme 2). The obtained polyamides were quantitative yields with inherent viscosity in the range of 0.52–0.96 dlg−1. All structural features of these resulting polyamides were verified by FT-IR (Figure 5). They exhibit characteristic absorption band of amide group at 3279.45 cm−1 is due to the – NH stretching indicates the disappearance of carboxylic group in polyamide and carbonyl stretching absorption band at 1654.45 cm−1.

Scheme 2. Synthesis of polyamides from 4 4ʹ bis(4-carboxy methylene) biphenyl and various aromatic diamines by Yamazaki’s phosphorylation method.

### 3.4 Solubility of polyamide

The solubility was qualitatively determined by the dissolution of 1 mg of polymer sample in 9 mg of solvent (10 wt%) at room temperature or an elevated temperature, and the results are summarized in Table 1. Almost all polyamides were readily soluble in polar solvents such as NMP, N, N-dimethyl formamide, DMAc, DMSO, Pyridine, and m-cresol at room temperature. These new polyamides exhibited higher solubility than the conventional aromatic polyamides methylene spacer group in the main chain of the polymers, as shown in Table 2.

**Table 2.** Yield and inherent viscosity of polyamides (PA-1 to PA-5)

Sr. no.	Polymer code	Diamine	Yield (%)	Inherent viscosity ήinh(dL/g)
1.	PA-1	ODA	93	0.96
2.	PA-2	MDA	95	0.56
3.	PA-3	SDA	97	0.70
4.	PA-4	DAP	94	0.60
5.	PA-5	TPTPDA	96	0.52

a) Polymerization was carried out with 1 mmol each of 4 4ʹ bis (4-carboxy methylene) biphenyl and various aromatic diamine.

b) 4 4ʹ bis (4-carboxy methylene) biphenyl.

c) Measured with 0.5% (w/v) polymer solution in DMF at 30 ± 0.1°C.

The solubility studies also revealed that solubility of polyamides enhances due to the incorporation of methylene spacer group at 4 4ʹ position on biphenyl as comparably to the spacer group present at 2 2ʹ position on biphenyl in which 4 4ʹ substituent at biphenyl decreases interchain interaction and increase free volume in polymer backbone than that of 2, 2ʹ substituent at biphenyl [23].

The X-ray studies revealed that all the polymers were completely amorphous in nature. Thus, the amorphous nature of these polymers was reflected in their excellent solubility and this could be attributed to the incorporation of methylene spacer group at 4, 4ʹ position on biphenyl unit along the polymer backbone.

### 3.5 Thermal properties

**Figure 6.**
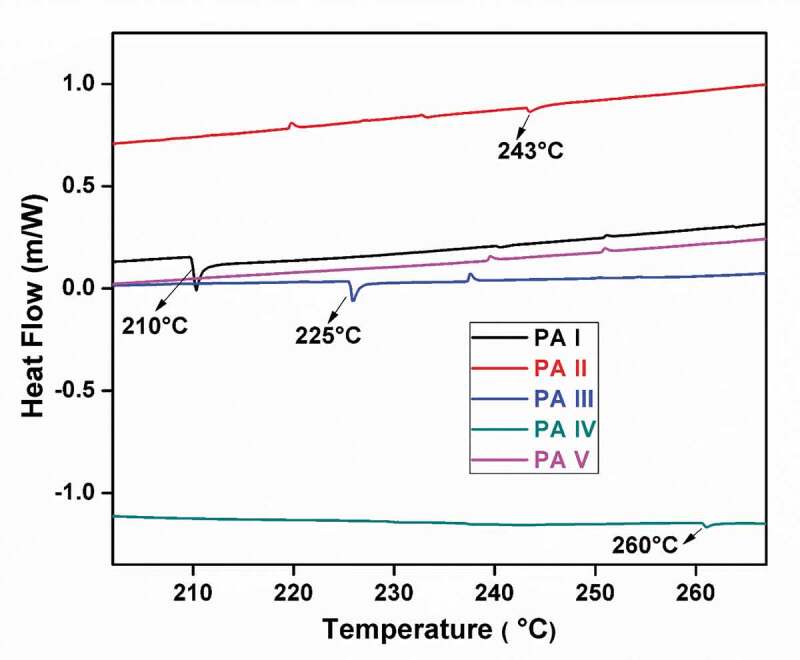
DSC curves of polyamides, PA-1 to PA-5

**Figure 7.**
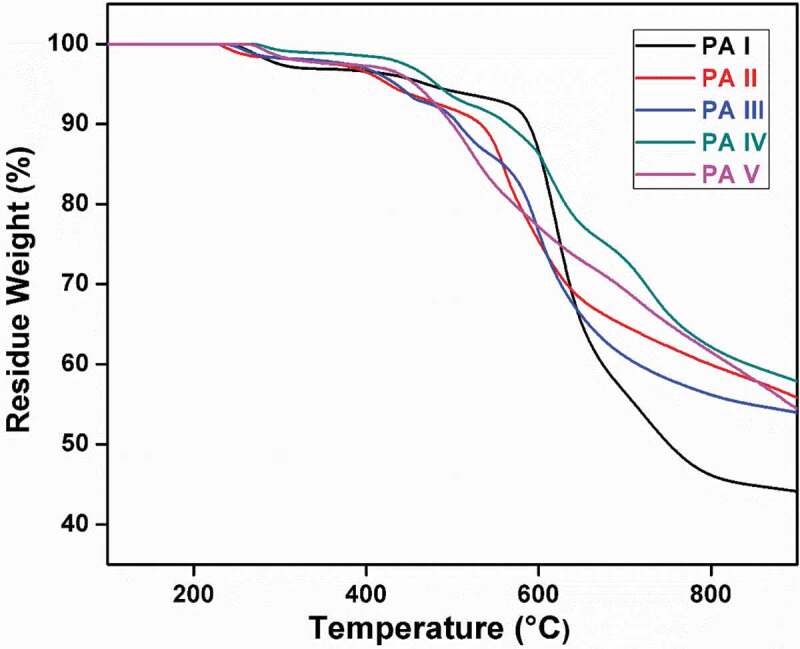
TGA curves of polyamides, PA-1 to PA-5

**Figure 8.**
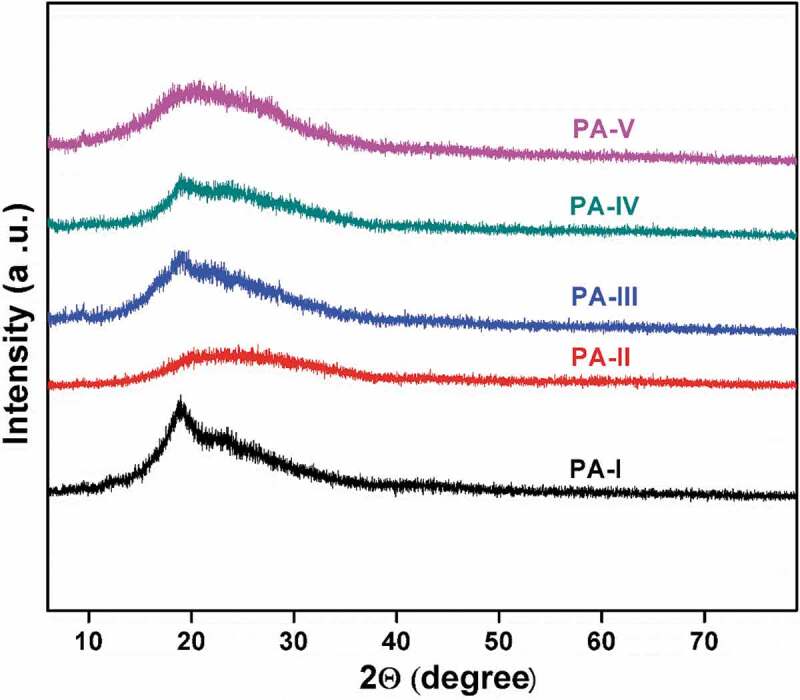
XRD curves of polyamides, PA-1 to PA-5

The thermal behavior and stability of the prepared polyamides were evaluated by means of TGA and DSC, and the results are summarized in Table 3. The DSC curves are shown in Figure 6Figure 7 and 8, whereas TGA curves of polyamides PA-1 to PA-5 are shown in Figure 5. These polyamides had a glass transition temperature in the range of 210–261°C. The PA-5 which did not show glass transition temperature Tg value due to the rigidity effect of tetraphenyl bulky pendant group attached to hetrocyclic thiophene moiety in polymer backbone. TGA in air was used for the investigation of the thermal stability of the polymers. The initial decomposition temperature was in the range of 247–230°C and 10% weight loss at temperature (T10) were in the range 497–597°C, respectively.

**Table 3.** Thermal propertiesa of polyamides

Polymer code	bTi (°C)	cT10% (°C)	dTg (°C)	Residual wt. % at 800°C
PA-1	238	588	210	46.13
PA-2	229	532	243	59.90
PA-3	240	507	225	56.17
PA-4	271	564	260	62.14
PA-5	265	499	-	61.40

a Thermogravimetric analysis was conducted at a heating rate of 10°C/min. under nitrogen atmosphere.

b Ti:- Temp at which weight loss initiated.

c T10%:- Temp at which 10% weight loss was observed.

d Tg:- Determined by DSC measured at a heating rate 20°C/min. under nitrogen atmosphere.

Structure-thermal property correlations based on the obtained carbonized residue (char yield) led to several interesting marks:

The properties of the polyamides depend not only on the structural composition but also on regularity of structure. All the polyamides begin to decompose at about 229°C. The TGA of polyamides (PA-I to PA- V) shows T10% weight loss temperature of the polyamides at 588 °C (PA-I), 532°C (PA-II), 507°C (PA-III), 564°C (PA-IV), 499°C (PA-V), and the amount of carbonized residue (char yield) value was 46.13% (PA-I), 59.90% (PA-II), 56.17% (PA-III), 62.14%(PA-IV), and 61.40% (PA-V) at 800 °C, respectively, as shown in Table 3.

The polyamides PA-III and PA-V contain sulfone linkages and heterocyclic thiophene unit in the polymer backbone, respectively. These polyamides show lower T10% weight loss residue temperature than the other polyamides. This might be attributed to the nature of weak bond between carbon and sulphur (C-S) and easy degradation in heating process.

The amount of carbonized residue (char yield) value at 800°C of polyamides containing pyridine (PA-IV) and thiophene (PA-V) moiety are more than the other polyamides containing spacer methylene (PA-II), sulfone linkages (PA-IV) and flexible ether (PA-I) moiety, due to the rigid or stiffness nature of heterocyclic ring. A higher order of thermal stability is achieved in these polyamides. Strong intermolecular binding and chain stiffness are also contributing factors for these polyamides.

Overall, the heterocyclic ring containing polymers exhibited better thermal stability than their analogues containing bridging sulfone, spacer methylene and flexible ether groups or moiety.

Comparative Thermal Stability Study of Polyamides containing Biphenyl Unit (PA-1) with other Biphenyl unit having Flexible, Spacer and Pendant groups

On comparing as shown in the above figure, the thermal stability of polyamides (PA-01; biphenyl unit, methylene linkages and ODA unit) with respect to T10% of the structurally related polyamides containing biphenyl unit with Pendant Flexible, Spacer groups in the polymer chains.

The order of thermal stability of biphenyl is as follows: The PA-01 (Biphenyl unit, Methylene linkages and ODA) polyamides (588°C) > Two -CF3 and Binaphthyl unit containing polyamides (549°C) > polyamides based on Pendant Phenyl Group (polyamide[> Two Pendant Methyl group cantered Polyamides (494°C) [30] > Two -CF3 Group containing Polyamides (490°C) > Two -CF3 and four pendant Methyl group covered Polyamides [24–27].

From the above thermal data study, we have concluded as the insertion of the Two -CF3 group and increase in the number of Pendant Methyl group reduces the thermal stability of Polyamides [27]. But if inserting the Pendant Phenyl or Binaphthyl unit with -CF3 group will reduce the solubility of polyamides. Hence, in our research work incorporation of methylene spacer group at 4 − 4ʹ position on the biphenyl unit indicates good thermal stability and solubility of polyamides.

## 4. Conclusion

A new 4 4ʹ bis(4-carboxy methylene) biphenyl monomer was successfully synthesized via fridal craft acylation reaction and willegrdot reaction with good yield. Aliphatic aromatic polyamides were synthesized from 4 4ʹ bis(4-carboxy methylene) biphenyl monomer with various aromatic diamines by Yamazaki phosphorylation reaction. Prepared polymers appeared amorphous in nature with good solubility in many aprotic solvents. Incorporation of methylene spacer group in polymer backbone increases the solubility and thermal stability of polymers.

From the above result, it was confirmed that the introduction of the methylene spacer group at 4 4ʹ position on biphenyl into the polymer backbone was highly effective for the preparation of soluble aromatic polyamides having both high Tg and high T10 value than the polymers derived from 2 2ʹ substituent biphenyl unit. The polymer we have developed could be used as a high-performance polymeric material.
